# Incidence and predictors of COPD mortality in Uganda: A 2-year prospective cohort study

**DOI:** 10.1371/journal.pone.0246850

**Published:** 2021-02-11

**Authors:** Patricia Alupo, Adaeze C. Wosu, Abdallah Mahofa, Levicatus Mugenyi, Daniel Semakula, Winceslaus Katagira, Bruce Kirenga

**Affiliations:** 1 Makerere University Lung Institute, Makerere University College of Health Sciences, Kampala, Uganda; 2 Department of Epidemiology, Johns Hopkins Bloomberg School of Public health, Baltimore, Maryland, United States of America; 3 Department of Medicine, Makerere University College of Health Sciences, Kampala, Uganda; BronxCare Health System, Affiliated with Icahn School of Medicine at Mount Sinai, NY, USA, UNITED STATES

## Abstract

**Background:**

Data is lacking on outcomes among COPD patients in sub-Saharan Africa. The objective of the study was to assess the incidence and predictors of mortality among COPD patients enrolled in the Uganda Registry for Asthma and COPD.

**Research question:**

What is the Incidence and predictors of mortality among COPD patients in Uganda?

**Study design and methods:**

Individuals with a diagnosis of COPD at six hospitals in Uganda were enrolled into the registry, and followed every six months. Mortality was ascertained through post-mortem reports and verbal autopsies. Mortality rates (MR), mortality rate ratios (MRR), and hazard ratios (HR) were computed to assess associations between socio-demographic, behavioural, and clinical characteristics at enrolment into the registry and mortality up to two years after.

**Results:**

We enrolled 296 COPD patients. Median age was 60 years, and 51·3% were male. The overall mortality rate was 95·90 deaths/1000 person-years. COPD severity by post-bronchodilator FEV_1_ was the strongest risk factor for mortality. Compared to stage 1, adjusted hazard ratios were as follows for stage 4: 9·86 (95%CI: 1·70–57·14, p = 0·011), stage 3: 6·16 (95%CI: 1·25–30·32, p = 0·025), and stage 2: 1·76 (95%CI: 0·33–9·48, p = 0·51). Underweight patients had a higher incidence of mortality compared to normal weight patients (MRR: 3**·**47 (95%CI: 1·45–8·31, p = 0·0026).

**Conclusion:**

Among COPD patients in Uganda, two-year mortality is high, and disease severity at baseline was the strongest risk factor for mortality. Our findings suggest the need for early, accurate, diagnosis and management of COPD, to potentially improve survival.

## Introduction

An estimated 210 million people suffer from Chronic Obstructive Pulmonary Disease (COPD) globally. COPD is projected to be the third leading cause of death in 2020, and the substantial number of people suffering from COPD is expected to increase [1, 2]. A preventable chronic airway disease characterized by progressive airflow limitation, COPD is associated with airway inflammation [3]. The airflow limitation is irreversible, and patients commonly present with symptoms of dyspnoea, cough and sputum production in the background of exposure to cigarette smoke or noxious stimuli. COPD is punctuated with episodes of worsened symptoms that are termed as exacerbations. Patients may be asymptomatic, with clinically significant COPD starting from GOLD stage II [3], and most patients present when a lot of damage has already been done.

Globally, it is estimated that 3·17 million deaths were caused by COPD in 2015, which is about 5% of all deaths globally. Cigarette smoking has been established as the most important risk factor for COPD in high-income countries, but non-traditional risk factors such as household air pollution or bio-mass exposure have since been identified in low- and middle-income countries (LMICs) [4, 5]. COPD has thus evolved to become a disease of smokers and non-smokers alike, with a prevalence of 3% - 11% in lifelong non-smokers globally [6, 7].

Most data on morbidity and mortality from COPD come from high-income countries, even though 90% of COPD attributable deaths occur LMICs like Uganda [8]. Studies conducted so far have found a significant burden of COPD in Uganda, with prevalence ranging from 6·1%- 16·8% among rural participants, and 1·5% in urban participants [9, 10]. In Uganda, COPD has been found to occur in a significantly younger population, with one study finding the highest prevalence among people aged 30 to 39 years [5, 6]. Previous studies, most from high income countries, have found varied factors to be associated with COPD mortality; these factors include low forced expiratory volume in 1 second (FEV_1)_, cardiovascular diseases, cancer [11, 12], as well as low forced vital capacity (FVC) [13, 14].

There is paucity of data on COPD mortality and predictors of mortality in LMICs, particularly countries within sub-Saharan Africa. Most data used to guide clinical practice in this setting has been generated from high-income countries. The aim of the current study was to assess the incidence and predictors of COPD mortality in Uganda.

## Methods

### Design and setting

This was a prospective cohort study nested within the Uganda Registry for Asthma and Chronic Obstructive Pulmonary Disease (URAC).

In August 2013, the registry began enrolling consecutive physician-diagnosed asthma and COPD patients attending chest clinics of six tertiary hospitals across Uganda. The URAC was established in six tertiary hospitals in Uganda, namely Mulago National Referral Hospital, Mbale Regional Referral Hospital, Hoima Hospital, Arua hospital, and Gulu Hospital. Patients that came to the facilities with chest symptoms underwent clinical evaluation as per the standard of care and those that had history and clinical findings in keeping with COPD underwent spirometry. For the current study, we only considered COPD patients who presented to the Mulago National Referral Hospital URAC site. This is currently the only national referral hospital in Uganda, thus patients referred from across the country present here. Although the patient population that presents at this facility may be subject to selection bias, we believe the heterogeneity of the patient population that was seen is representative of the larger population of Uganda.

At baseline (i.e., the date of enrolment into the registry), each subject that met the clinical criteria for COPD was requested to undergo spirometry testing according to American Thoracic Society/European Respiratory Society (ATS/ERS) guidelines using a Pneumotrac® spirometer with Spirotrac® V software (Vitalograph Ltd., Buckingham, UK). Lung function was quantified using predicted parameters based on NHANES III models for African-Americans [15]. Parameters collected were forced vital capacity (FVC), Forced Expiratory Volume in I second (FEV_1_), FEV % predicted and FEV_1_/FVC ratio which was used as part of the diagnosis of COPD. The spirometer was calibrated daily to ensure accuracy of the measurements.

The process of diagnosing COPD was as follows; First, a COPD diagnosis was made clinically, with a clinician evaluating the patient symptoms, physical findings and exposures they had in their background, like cigarette smoking and biomass use. The Symptoms evaluated included cough, chest pain, sputum, wheezing, shortness of breath and body swelling. After a clinical evaluation by the physician, individuals that had a clinician diagnosis of COPD then underwent spirometry. Finally, the spirometry diagnosis of COPD was made if the subject had a post bronchodilator FEV_1_/FVC of < 0.7. A test was considered acceptable if it met the quality checks, which included a good start, rise to peak, smooth descent with absence of cough in the first 1 second and good end of test. For the test to meet the repeatability criteria, the subject had to have at least two acceptable tests with FVC and FEV_1_ within 150mls of each other. In addition, we collected information on patient’s socio-demographics, behavioural, and clinical profiles using a standardised clinical record form (CRF) developed by our team (S1 and S2 Appendices). More detailed Information on risk factors that subjects were exposed to was also collected to concretize the COPD diagnosis as per GOLD guidelines. Subjects were requested to return every six months for follow-up.

At follow-up visits, information was collected on respiratory symptoms, vital signs, anthropometry, medications, visits to health facilities and hospitalizations since the last visit. In addition, spirometry was performed and nurses reminded patients of their next follow-up appointments. Where necessary, follow-up information was collected by phone. Registry medical officers prescribed and advised treatment based on local practice guidelines. Patients continued to obtain medications from hospital pharmacies or other health centres, and attended regular chest clinic visits as requested by attending physicians. The registry did not provide medications and incentives to participants.

The main outcome was all-cause mortality. For patients who died in hospital, mortality information was obtained from hospital charts or post-mortem reports. For patients who died outside the hospital, a verbal autopsy form was used to collect information about the circumstances and possible cause of death from relatives or caretakers of the deceased. The verbal autopsy form was developed using WHO guidelines [16].

### Ethical considerations

Ethical approval for URAC was obtained from the Mulago Hospital Research and Ethics Committee and the Uganda National Council for Science and Technology (MREC number 461). All COPD patients provided written informed consent prior to enrolment into URAC.

### Statistical analysis

The information included in this manuscript is based on subjects enrolled between August 2013 to April 2016. First, we assessed distributions of baseline socio-demographic (sex, age), behavioural (tobacco smoking, exposure to biomass) and clinical profiles (COPD severity, HIV status, previous tuberculosis treatment, hypertension, medication use, blood oxygen saturation (SPO_2_), body mass index), and summarized them using counts and percentages, means (and standard deviations), and medians (and interquartile ranges) as appropriate. We computed mortality rates (per 1,000 person-years) over two years of follow-up (using total number of deaths as the numerator and total follow-up time in years as the denominator); and corresponding mortality rate ratios, and 95% confidence intervals (CI).

We fitted unadjusted and adjusted Cox regression models to assess relative hazards of mortality within two years post-enrolment. The time origin was the date on which each participant was enrolled into the study, as such there was no late entry. Selection of the final multivariable Cox regression model was guided by *a priori* hypotheses about biological plausibility, clinical relevance, assessment of multicollinearity among selected factors, and comparisons of Akaike Information Criteria (AIC) within candidate final models. Furthermore, we avoided relying on a p-value cut-off for selecting risk factors, as potentially important factors were likely to lack statistical significance in our modest sample size. We assessed whether the proportional hazards assumption held using a combination of visual comparisons (via Kaplan-Meier curves) and Schoenfeld tests. From the Schoenfeld test, a non-significant relationship (a random pattern) between time and the residuals supports the assumption of proportionality. The graphs for survival suggested proportionality for all variables except sex over the course of the two years. Specifically, relative hazards of mortality changed substantially after the first year of follow-up. As such, and due to substantial differential attrition by sex after the first year of follow-up, we conducted additional analysis in which observations were censored at 1-year post-enrolment. The proportional hazards assumption held for all variables when outcome assessment was limited to 1-year follow-up. All statistical tests were two-sided with alpha set at 0·05. Analyses were performed using STATA 14 (Stata Corp, College Station, TX, USA).

## Results

### Baseline characteristics of COPD patients in the URAC registry

Between August 2013 and April 2016, a total of 296 patients with a physician diagnosis of COPD were enrolled in the URAC registry (51·3% male, median age was 60 years (interquartile range: 44–70)). The majority of patients were enrolled at Mulago Hospital Chest Clinic, Pulmonary Ward or Accident and Emergency Ward (n = 212), and Nsambya Hospital (n = 30). The remaining 54 patients were enrolled from other hospitals and clinics. While all patients completed a pre-bronchodilator spirometry at baseline, only 225 (76·0%) successfully completed post-bronchodilator FEV_1_/FVC readings (Table 1).

**Table 1:** Characteristics of COPD patients at baseline (n = 296).

Characteristics	N (%)
Female	144 (48·7%)
**Age,** *years*	
Median (IQR)	60 (44, 70)
**Marital Status**	
Single	33 (11·2%)
Married	173 (58·4%)
Separated	32 (10·8%)
Widowed	58 (19·6%)
**Employed,** *yes*	140 (47·3%)
**Education,** *highest level*	
None	41 (13·9%)
Some/Completed Primary	141 (47·8%)
Some/Completed Secondary	64 (21·7%)
Tertiary	49 (16·6%)
**Respiratory Symptoms,** *yes*	
Cough	261 (88·1%)
Sputum	210 (70·9%)
Wheezing	238 (80·4%)
Shortness of breath	273 (92·2%)
Chest pain	216 (72·9%)
**Lung Function (baseline post-bronchodilator FEV**_**1**_**/FVC ratio), %**	
Median (IQR)	61 (49, 68)
*Missing*	71 (23·9%)
**Severity of COPD by GOLD stage (based on baseline post-bronchodilator FEV**_**1**_**)**	
Stage 1 (FEV_1_ ≥ 80%)—mild	71 (23·9%)
Stage 2 (FEV_1_ 50–79%)—moderate	86 (29·1%)
Stage 3 (FEV_1_ 30–49%)—severe	49 (16·6%)
Stage 4 (FEV_1_ < 30%)–very severe	19 (6·4%)
*Missing*	71 (23·9%)
**Medications,** *yes*	
Salbutamol inhaler	144 (48·7%)
Inhaled corticosteroids	30 (10·1%)
Combination inhalers (steroid, LABA)	25 (8·5%)
Leukotriene modifiers	13 (4·39%)
Any medications for COPD	153 (51·69%)
**Risk factors and co-morbid conditions**	
History of tobacco smoking	
Never smoked tobacco	169 (57·1%)
Former tobacco smoker	89 (30·1%)
Current tobacco smoker	38 (12·8%)
Use of biomass†, *yes*	274 (92·6%)
Ever been treated for TB, *yes*	62 (20·9%)
HIV status	31 (10·4%)
Positive	31 (10·5%)
Negative	241 (81·4%)
Unknown	24 (8·1%)
Nasal congestion or rhinorrhea, *yes*	156 (52·7%)
Heart burn, *yes*	168 (56·7%)
Body mass index (BMI), kg/m^2^, (median and IQR)	21·4 (19·1, 25·0)
Underweight (BMI < 18·5 kg/m^2^), %	58 (19·7%)
Normal weight (BMI 18·5 to 24·99 kg/m^2^), %	165 (55·9%)
Overweight (BMI 25kg/m^2^ to 29·99 kg/m^2^), %	42 (14·2%)
Obese (BMI ≥ 30 kg/m^2^), %	30 (10·2%)
Hypertensive, i·e·, SBP/DBP cutoff 140/90 mmHg	101 (34·1%)
SPO_2_, % (median and IQR)	96 (92, 98)
**Number of moderate/severe exacerbations (within the past one year)**	
< 3	194 (65·8%)
≥ 3	101 (34·2%)

Abbreviations used in the table: BMI: body mass index; COPD: chronic obstructive pulmonary diseases; CS: Corticosteroid; DBP: diastolic blood pressure; FEV_1_: forced expiratory volume in the first second; FVC: forced vital capacity; IQR: interquartile range; LABA: long acting beta2 agonist; SBP: systolic blood pressure; TB: tuberculosis; SPO_2_: peripheral capillary oxygen saturation, %.

Biomass refers to wood and charcoal (typically used for cooking purposes).

Respiratory symptoms were highly prevalent at baseline, with 88·1% reporting cough, 70·9% sputum, 80·4% wheezing, 92·2% shortness of breath, and 72·9% chest pain (Table 1). With regard to known behavioural risk factors for COPD, 20·9% reported that they had ever been treated for tuberculosis, 42·9% identified as current or former smokers, and the majority of participants (92·5%) reported use of biomass—i.e., use of wood, or charcoal for domestic purposes. In terms of treatment and management of symptoms, a total of 48·7% reported use of salbutamol inhalers, 10·1% reported use of inhaled corticosteroids, and 8·5% reported use of combination inhalers (steroids and long-acting beta2-agonists (LABA)). No patient (0%) reported using long acting muscarinic antagonists (LAMAs) like Tiotropium because they were largely unavailable in the country at time of the study. Among the 225 participants who completed acceptable post-bronchodilator spirometry readings at baseline, median post-bronchodilator FEV_1_/FVC reading was 61%, with an interquartile range of 49% to 68%.

### Incidence of mortality

**Fig 1:**
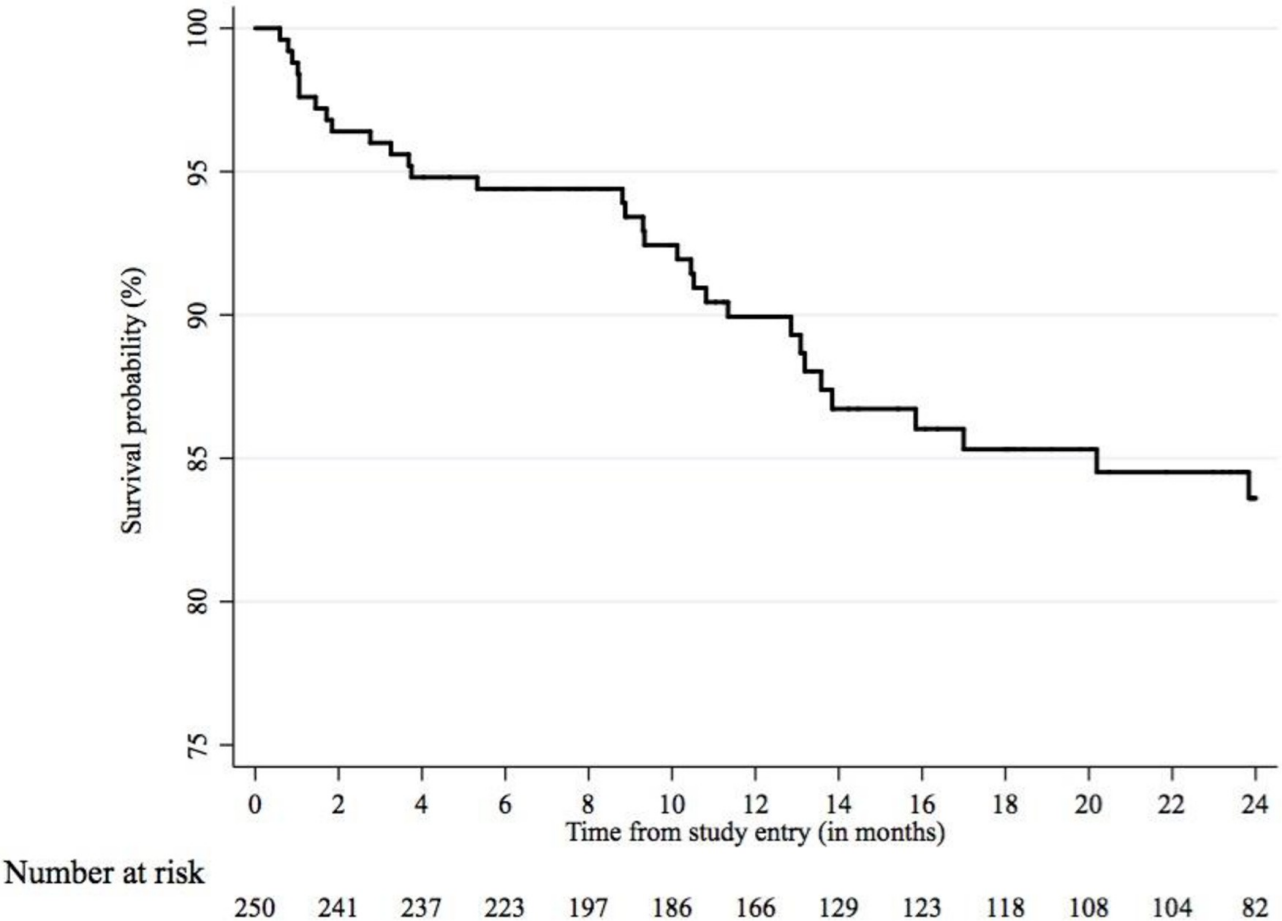
Survival probability among COPD patients over 2 years (n = 250).

Fig 1 illustrates the probability of survival over two years. Deaths were registered for a total of 33 (11·2%) patients, translating into an overall mortality rate of 95·90 deaths per 1000 person-years. Cumulative incidence of death (Kaplan-Meier estimate) was 16·39% over two years.

### Predictors of mortality

In Table 2, we present mortality rates (MR) and mortality rate ratios (MRR) according to patients’ characteristics.

**Table 2:** Mortality rates and mortality rate ratios among COPD patients, stratified by baseline characteristics.

Characteristic	Number of COPD patients, N, % (column)	Number of deaths N (row)	Total follow-up time (in person-years)	Mortality rateᶲ (Deaths /1000 p-years)	Mortality rate ratio, (95% CI)	p-value
**Overall**	250	32	333·67	95·90	**--**	**--**
**Sex**						
Female	123 (49·2%)	14	175·26	79·88	**1·0 (Ref)**	
Male	127 (50·8%)	18	158·41	113·63	1·42 (0·67, 3·09)	0·32
**Age in years**						
<35	33 (13·2%)	3	47·84	62·71	**1.0 (Ref)**	
35–44	25 (10·0%)	1	35·43	28·22	0·45 (0·01, 5·60)	0·54
45–54	43 (17·2%)	8	56·36	141·95	2.26 (0·54, 13·24)	0·23
55–64	55 (22·0%)	7	73·89	94·73	1·51 (0·34, 9·05)	0·58
65+	94 (37·6%)	13	120·15	108.20	1·73 (0·47, 9·44)	0·41
**Severity of COPD by GOLD stage (based on baseline post-bronchodilator FEV**_**1**_**)**						
Stage 1 (FEV_1_ ≥ 80%)—Mild	59 (23·6%)	3	79·95	37·52	**1·0 (Ref)**	
Stage 2 (FEV_1_ 50–79%)—Moderate	73 (29·2%)	5	97·16	51·46	1·37 (0·27, 8·83)	0·69
Stage 3 (FEV_1_ 30–49%)—Severe	40 (16·0%)	8	52·32	152·91	4·07 (0·97, 23·85)	0·032
Stage 4 (FEV_1_ < 30%)–Very Severe	17 (6·8%)	6	21·55	278·37	7·41 (1·58, 45·84)	0·0047
*Missing*	61 (24·4%)					
**History of tobacco smoking**						
Never smoked tobacco	147 (58·8%)	15	207·65	72·24	**1·0 (Ref)**	
Former tobacco smoker	75 (30·0%)	11	92.93	118.36	1.64 (0.68, 3.82)	0.22
Current tobacco smoker	28 (11·2%)	6	33.09	181.47	2.51 (0.80, 6.85)	0.08
**Exposure to biomass**						
No	18 (7·2%)	3	18·16	165·18	**1·0 (Ref)**	
Yes	232 (92·8%)	29	315·51	91·92	0·56 (0·17, 2·85)	0·35
**Use of any COPD medications (salbutamol inhaler, inhaled corticosteroids, Combined CS/LABA, Leukotriene modifiers**						
No	120 (48·0%)	13	158·15	82·0	**1·0 (ref)**	0·44
Yes	130 (52·0%)	19	175·16	108·47	1·32 (0·62, 2·91)	
**HIV status**						
Negative	202 (80·8%)	25	262·58	95·21	**1·0 (Ref)**	
Positive	27 (10·8%)	6	32·01	187·44	1·97 (0·66, 4·91)	0·16
Unknown	21 (8·4%)	1	39·08	25·58	0·27 (0·01, 1·64)	0·16
**Previous TB treatment**						
No	197 (78·8%)	24	264·21	90·83	**1.0 (Ref)**	
Yes	53 (21·2%)	8	69·46	115·17	1·27 (0·49, 2·92)	0·55
**Hypertensive (140/90 mmHg criteria)**						
No	88 (35·2%)	24	216·79	110·71	**1·0 (Ref)**	
Yes	162 (64·8%)	8	116·88	68·45	0·62 (0·24, 1·42)	0·24
**SPO**_**2**_**. %**						
≥ 90	248 (83·8%)	21	76·84	273·32	**1·0 (Ref)**	
< 90	45 (15·2%)	9	159·30	56·50	2·07 (0·84, 4·72)	0·082
**BMI categories**						
Underweight (BMI < 18·5 kg/m^2^)	53 (21·3%)	13	59·32	219·16	**3**·**47 (1·45, 8·31)**	0·0026
Normal weight (BMI 18.5 to 24.99 kg/m^2^)	136 (54·6%)	12	189·99	63·16	**1**·**0 (Ref)**	
Overweight (BMI 25kg/m^2^ to 29.99 kg/m^2^)	35 (14·1%)	4	46·75	85·57	1·35 (0·32, 4·47)	0·59
Obese (BMI ≥ 30 kg/m^2^)	25 (10·0%)	3	36·46	82.·28	1·30 (0·24, 4·83)	0·66
**Number of moderate or severe exacerbations at baseline (within one year prior to baseline)**						
<3	166 (66·4%)	20	223·15	89·63	**1·0 (Ref)**	
≥ 3	83 (33·2%)	12	108·52	110·57	**1·23 (0·55, 2·65)**	0·56

These MR and MRR were from sub-group analysis of the 84·5% participants (250/296) who returned for at least one subsequent clinic visit, or whose survival status could be ascertained at a later time. Severity by post-bronchodilator FEV_1_ was prognostic for mortality. Specifically, MRR and corresponding 95% CI were as follows for GOLD stage 3 [MRR = 4·07 (0·97, 23·85)] and for GOLD stage 4 [MRR = 7·41 (1·58, 45·84)]. We observed higher, but not statistically significant MR per 1,000 person-years for males compared to females (113·63 vs. 79·88), as well as for former and current smokers (118.36 and 181.47) compared with those who have never smoked (72·24).

In Table 3, we present results of the Cox regression models over two years.

**Table 3:** Unadjusted and multivariable adjusted hazard ratios for mortality among COPD patients across two years.

Characteristics	Crude			Adjusted		
	HR	95% CI	P-value	HR	95% CI	P-value
**Sex:** Female vs. Male	0·71	0·35, 1·42	0·33	0.56	0·18, 1·76	0·32
**Age in years**	1·01	0·99, 1·03	0.41	1·01	0·98 1·05	0.35
**Severity of COPD by GOLD stage (based on baseline post-bronchodilator FEV**_**1**_**)**						
*Stage 1*	REF			REF		
*Stage 2*	1·36	0·33, 5·76	0·67	1·76	0·33, 9·48	0·51
*Stage 3*	**4**·01	**1·06, 15·11**	**0·040**	**6·16**	**1·25, 30·32**	**0·025**
*Stage 4*	**7·20**	**1·80, 28·79**	**0**·**0050**	**9·86**	**1·70, 57·14**	**0·011**
**Hypertensive:** *Yes vs*. *No*	0·62	0·28, 1·37	0·24	0·56	0·18, 1·72	0·31
**BMI categories (kg/m**^**2**^**)**						
Normal weight (BMI 18·5–24·99 kg/m^2^)	REF			REF		
Underweight (BMI < 18·5 kg/m^2^)	**3·73**	**1·68, 8·30**	**0·0010**	1·91	0·61, 6·01	0·27
Overweight/obese (BMI ≥25kg/m^2^)	1·46	0·56, 3·84	0·44	1·52	0·43, 5·41	0·52
**History of tobacco smoking**						
Never smoked tobacco	REF			REF		
Former tobacco smoker	1·63	0·75, 3·54	0·22	0·37	**0**·10, 1·38	0·14
Current tobacco smoker	2·55	0·988, 6·58	0·053	1·22	**0**·30, 4·91	0·78
SPO_2_ level: *<90% vs*. *90% (Ref)*	2·13	0·98, 4·66	0·057	1·34	**0**·45, 3·98	0·60

Multivariable adjusted model included all variables in the table; HR: Hazards Ratio.

Overweight and obese BMI categories were collapsed due to few events within the obese category.

**Fig 2:**
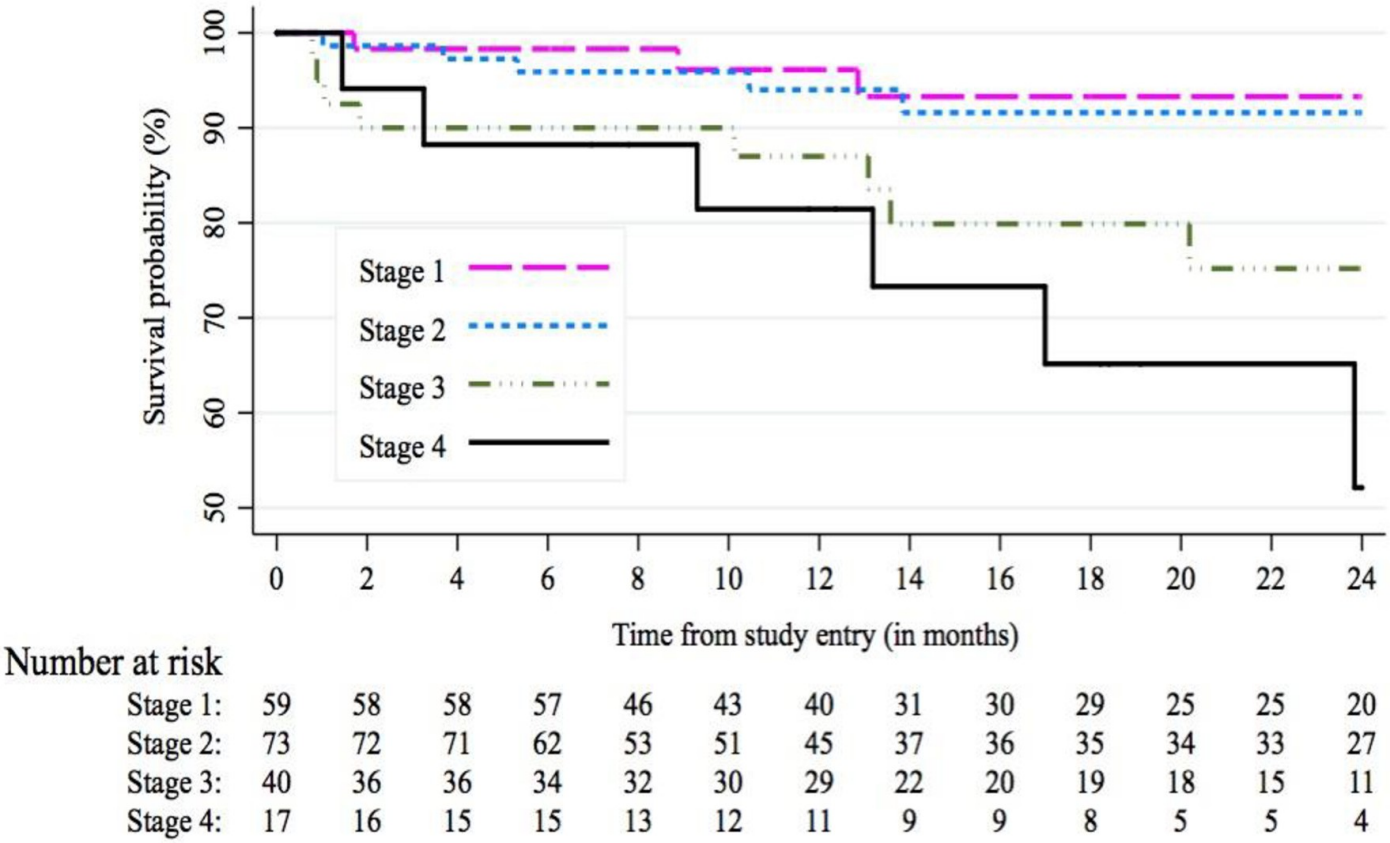
Survival probability according to COPD severity (staging by baseline post-bronchodilator FEV_1_).

**Fig 3:**
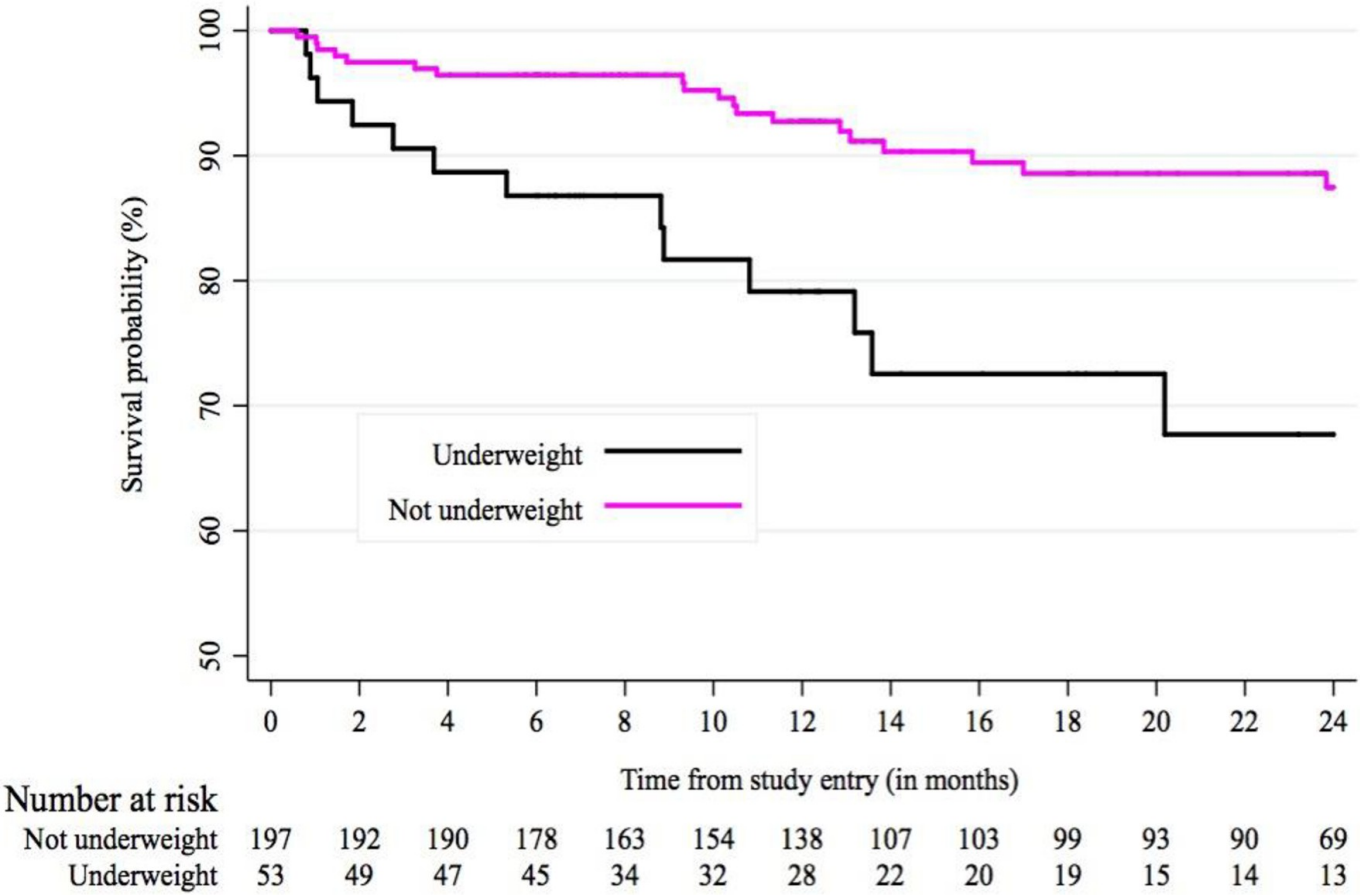
Survival probability according to underweight status.

Results of the Schoenfeld test showed that the proportional hazards assumption held for all factors in the final model except sex (*rho* = 0·607, p = 0·0042), indicating an increasing log-hazard ratio over time. In the multivariable Cox regression model, severity by COPD GOLD stage at baseline was the strongest predictor of mortality. Compared to GOLD stage 1, HR for GOLD stage 3 was 6·16 (95% CI: 1·25, 30·32, p = 0·025), and for GOLD stage 4, HR = 9·86 (95% CI: 1·70, 57·14, p = 0·011). In the unadjusted model, being underweight at baseline was associated with higher rate of mortality, compared with being of normal weight at baseline; however, the association was not statistically significant in the multivariable model (HR = 1·91 (95% CI: 0·61, 6·01, p = 0·27). When we added use of any COPD medications at baseline into the model, we observed higher relative hazard of death with medication use (HR = 2·72 (95% CI: 0·97, 7·65, p = 0·057), without substantial changes to the hazard ratios of the other variables in the model (not shown in tables). Figs 2 and 3 show Kaplan-Meier curves for survival probability by COPD GOLD stage and underweight (log-rank test for both comparisons were p<0·0001). A sub-analysis of only participants with a self-reported HIV positive or negative status (n = 172), and controlling for previous tuberculosis treatment suggested higher HR of death among HIV positive individuals compared to HIV negative individuals (HR: 5·09; 95% CI: 1·43, 18·08, p = 0·012). Due to limited variation in biomass use (92·6% were biomass users), we were unable to examine the association of this variable with mortality in the adjusted Cox models.

In Table 4, we present results of Cox regression analysis with all observations censored after one year of follow-up.

**Table 4:** Multivariable adjusted hazard ratios for mortality among COPD patients, for the first year under observation.

Characteristics	Period (baseline to year 1)		
	HR	95%CI	P-value
**Sex:** *Female vs*. *Male (Ref)*	0·18	0·021, 0·64	0·014
**Age in years**	1·02	0·98, 1·07	0.24
**Severity of COPD by GOLD stage (based on baseline post-bronchodilator FEV**_**1**_**)**			
*Stage 2 vs*. *Stage 1*	2·59	0·25, 26·48	0·42
*Stage 3 vs*. *Stage 1*	9·59	1·03, 89·01	0·047
*Stage 4 vs*. *Stage 1*	6·67	0·53, 84·11	0·14
**Hypertensive:** *Yes vs*. *No (Ref)*	0·49	0·11, 2·08	0·13
**BMI categories (kg/m**^**2**^**)**			
*Normal weight (BMI 18·5–24·99 kg/m*^*2*^*)*	REF	**--**	**--**
*Underweight (BMI < 18·5 kg/m*^*2*^*)*	1·86	0·49, 7·12	0·36
*Overweight/obese (BMI ≥25kg/m*^*2*^*)*	0·64	0·072, 5·67	0·67
**Smoking history**			
*Never smoked tobacco*	REF	--	--
*Former tobacco smoker*	0·25	0·05, 1·19	0·082
*Current tobacco smoker*	0·81	0·17, 3·92	0·79
SPO_2_ level: *<90% vs*. *≥90% (Ref)*	2·37	0·65, 8·61	0·19

Multivariable adjusted model included all variables in the table; HR: hazards ratio.

Overweight and obese BMI categories were collapsed due to few individuals within the overweight category.

The proportional hazards assumption held for all variables, and women had a much lower relative hazard of death compared to men (HR: 0·18, 95% CI: 0·021, 0·64, p = 0·014). Moreover, although not statistically significant, hazards ratios were congruent with higher mortality by COPD GOLD stage 4, and by underweight status. We present baseline differences in men and women within S1 Table. Of note, at baseline, women had better median baseline lung function, were less likely to smoke tobacco, but reported more wheezing.

## Discussion

This is one of the first observational prospective studies evaluating mortality among COPD patients in Uganda. We assessed data for a total of 296 COPD patients. Median age was 60 years (interquartile range: 44–70). The cumulative incidence of death using Kaplan Meier estimates was 16·39% over the study period, and COPD GOLD stage severity (using baseline post-bronchodilator FEV_1_) was the strongest predictor of mortality, followed by underweight status. Mortality rates were 79·9/1000 person-years and 113·6/1000 person-years for females and males, respectively.

The cumulative incidence of death of 16.39% among COPD patients observed in our study was high, but comparable to the TORCH and UPLIFT study, and much lower than some study findings such as the BOLD study in South Africa. The TORCH study was a randomised double-blind clinical trial carried out across 444 centres in 42 countries including the United States of America, and countries in eastern Europe, western Europe and Asia-Pacific. The study evaluated the effect of long-acting beta agonists and inhaled corticosteroids on survival in COPD patients over a period of three years. All-cause mortality rate was 12·6% for the combination therapy group, 13·5% for the salmeterol group, 16.0% for the fluticasone group, and 15·2% for the placebo group. Although the study setting and design differed from ours (RCT vs cohort), and the population involved had largely been smokers, the mortality rates were comparable to our population [12]. Similarly, in the UPLIFT study, which was a randomised placebo control study evaluating long term effects of tiotropium therapy on COPD patients, patients were followed for a period of four years for primary end points of rate of decline of FEV_1_ before and after bronchodilation beginning at day 30, and secondary end points included mortality. They observed a mortality rate of 14·9% in the tiotropium group and 16·5% in the placebo group, though both of the above-mentioned studies did not evaluate or account for biomass exposure [17], which would be rare in the setting that participants were recruited from.

Conversely, our mortality rate was lower than that found in some studies such as the BOLD study in South Africa, where 107 patients with COPD were followed up for a period of 5 years and mortality rate was found to be 23% [18]. The difference in mortality with the BOLD study could have been the length of follow up, having evaluated their outcomes over a five-year follow-up period compared to the two years period for our study. Similarly, a longitudinal study conducted in the primary health care setting in Greece by Markos et al, in which 263 participants with COPD were followed up for two years, found a mortality rate of 27.9% [19]. While the study by Markos and colleagues had the same follow-up period as ours (two years), the higher mortality observed within their study could have been due to their older patient population (median age of 74 years vs. median age of 60 years in URAC). As with our study, the highest proportion of participants in both the BOLD study, and the study by Markos and colleagues had moderate COPD. Within the Ugandan setting, biomass exposure was the most ubiquitous exposure, with 92% of the participants having a history of biomass exposure, compared to cigarette smoking where only 41.2% of the participants had ever smoked. In contrast, the BOLD study had cigarette smoking as the predominant exposure among their participants, with 56.1% of them being current smokers and another 31.8% being ex-smokers. Similarly, the study by Markos *et al* also had cigarette smoking as the most prevalent exposure with 50.85% and 49.15% of the participants being current and ex-smokers respectively.

We observed a higher mortality rate for males than for females, though not statistically significant, similar to findings by Torres *et al*, who evaluated differences in COPD mortality between women and men across five countries (USA, Spain, Chile, Uruguay, Venezuela) where 265 females were enrolled between 1997 and 2006, and 272 matched males by region, BODE index and COPD stage enrolled and followed up for more than one year. The authors found higher all-cause mortality of 40% versus 18%, and respiratory-cause mortality of 24% versus 10% in males versus females respectively [20]. There are a number of possible explanations for this. As we observed, men were more likely to be at a later COPD GOLD stage than women and were more likely than females to be lost to follow-up after the first year, suggesting that men may be accessing care at a point when the disease process is much further along and thus may be more likely to deteriorate. The disease process may also be different overall in men and women, given that a combination of both tobacco smoking and biomass exposure could have an additive effect on the pathogenesis of COPD [21]. However Ringbaek *et al* who analysed mortality among 869 COPD patients seeking pension who were followed up for a mean period of 13·3 years found a higher standardized mortality rate in females than in males, with standardized mortality rates of 2·7 (2·5–3·0) in males compared to 4·8 (4·2–5·4) in females [22].

We noted important differences between male and female participants at baseline, with significantly more males having a tobacco smoking history compared to females (64·5% versus 20·1%), as well as greater COPD GOLD stage severity. After adjusting for covariates, males had significantly higher relative hazard of death compared to women in the first year, but not in the entire two-year period.

We found that the predictors with the greatest strengths of association after controlling for relevant covariates were GOLD stages of COPD severity, followed by underweight status. The association of being underweight with mortality is similar to findings in other studies highlighted in a meta-analysis by Yibin Guo and team [23]. This has been postulated to be due to impaired gaseous exchange, respiratory muscle weakness, impaired immune response, and loss of metabolically and functionally active fat-free mass in patients with low body mass [24]. Some studies have demonstrated other parameters as being stronger predictors of mortality, like exacerbation history [25]. Additionally, the BODE index (body mass index (B), airflow obstruction (O), dyspnoe (D), and exercise capacity (E)), has been demonstrated to be a better tool for measuring risk of mortality among these patients [26], but is challenging to use in clinical practice because of the requirement for a field exercise test.

We observed high prevalence of co-morbidity with cardiovascular disease (hypertension), which is in keeping with other studies globally that have found the most prevalent co-morbidity in COPD patients to be cardiovascular disease, though the direction of the association appeared to be counterintuitive, probably because they received treatment. Although we are unable to exhaustively examine biomass exposure as a risk factor due to lack of exposure variation in our sample, we believe that efforts to reduce biomass exposure, with as much effort as is done for smoking cessation, could also potentially lead to reduction of lung function decline among COPD patients, particularly in low and middle-income countries with high prevalence of household biomass usage.

The strengths of the study are that we used a prospective approach, and as such are able to establish temporality between the socio-demographic, behavioural, and clinical factors and the outcome. Our study provides seminal information on factors associated with mortality among COPD patients within Uganda.

Despite the prospective study design, our findings should be interpreted within the context of certain limitations. We experienced substantial attrition in number of patients returning for follow-up visits after the first year of the study, which limited statistical power to examine associations between the first and second year. We did not use a random sampling of COPD patients within the general population, but rather relied on a convenience sample of COPD patients who were visiting the health facilities and were willing to participate. Therefore, our findings are likely to have limited generalizability for patients seeking care outside of the hospital system. Due to our relatively small sample size, estimates are at times imprecise, particularly within the multivariable adjusted models.

## Conclusion

We have reported evidence on the incidence and the predictors of mortality among COPD patients in Uganda, which are more advanced disease by GOLD stage and low BMI. Preventative measures aimed at reducing biomass exposure, and early diagnosis by increasing awareness, establishment of diagnostic capacity in health facilities and screening those at risk in the population could potentially reduce incidence of COPD and capture those with early disease, and give opportunity for early intervention to reduce mortality. Among diagnosed COPD patients, non-pharmacological management approaches like diet that address BMI should be diligently incorporated into the management approaches of COPD patients in this setting to reduce mortality. More robust prospective studies are still needed in this setting to assess, as well as explain potential biological and social mechanisms from risk factors to mortality, putting into consideration factors such as exercise capacity, degree of dyspnoea, and biomarkers such as fibrinogen and troponins.

## Supporting information

S1 Table Comparison of baseline characteristics among men and women. (DOCX)

S1 Appendix (PDF)

S2 Appendix (PDF)
