# Supplementary material for: Incidence and predictors of COPD mortality in Uganda: A 2-year prospective cohort study
Source: PLoS One. 2021 Feb 11;16(2):e0246850. doi: 10.1371/journal.pone.0246850 (PMC7877567; doi:10.1371/journal.pone.0246850)
Supplement: S1 Table — (DOCX) [file pone.0246850.s001.docx]

| **S1 Table. Comparison of baseline characteristics among men and women** | | | |
| --- | --- | --- | --- |
| **Characteristics** | **Men**  **(n=152)** | **Women**  **(n=144)** | **p-value** |
| **Age,** *years* |  |  |  |
| Median (IQR) | 58 (43·5, 70) | 60 (45, 70) | 0·563 |
| **Marital Status** |  |  |  |
| Single | 19 (12·5%) | 14 (9·7%) | < 0·001 |
| Married | 108 (71·1%) | 65 (45·1%) |  |
| Separated | 13 (8·6%) | 19 (13·1%) |  |
| Widowed | 12 (7·8%) | 46 (31·9%) |  |
| **Employed,** *yes* | 91 (59·8%) | 49 (34·0%) | < 0·001 |
| **Education,** *highest level* |  |  |  |
| None | 6 (3·9%) | 35 (24·3%) | < 0·001 |
| Some/Completed Primary | 73 (48·0%) | 68 (47·2%) |  |
| Some/Completed Secondary | 39 (25·7%) | 25 (17·4%) |  |
| Tertiary | 33 (21·7%) | 16 (11·1%) |  |
| **Respiratory Symptoms,** *yes* |  |  |  |
| Cough | 134 (88·2%) | 127 (88·2%) | 0·992 |
| Sputum | 105 (69·1%) | 105 (72·9%) | 0·467 |
| Wheezing | 120 (78·9%) | 118 (81·9%) | 0·516 |
| Shortness of breath | 136 (89·5%) | 137 (95·1%) | 0·069 |
| Chest pain | 105 (69·1%) | 111 (77·1%) | 0·121 |
| **Lung Function (baseline post-bronchodilator FEV_1_/FVC ratio), %** |  |  |  |
| Median (IQR) | 58 (46, 65) | 64 (52, 69) | < 0·01 |
| *Missing* |  |  |  |
| **Severity of COPD by GOLD stage (based on baseline post-bronchodilator FEV_1_)** |  |  |  |
| Stage 1 (FEV_1_ ≥ 80%) - mild | 30 (26·6%) | 41 (36·6%) | 0·188 |
| Stage 2 (FEV_1_ 50 – 79%) - moderate | 43 (38·1%) | 43 (38·4%) |  |
| Stage 3 (FEV_1_ 30 – 49%) - severe | 27 (23·9%) | 22 (19·6%) |  |
| Stage 4 (FEV_1_ < 30%) – very severe | 13 (11·5%) | 6 (5·4%) |  |
| *Missing* | *39 (25·7%)* | *32 (22·2%)* |  |
| **Medications,** *yes* |  |  |  |
| Salbutamol inhaler | 62 (40·8%) | 82 (56·9%) | 0·005 |
| Combination inhalers (steroid, LABA) | 11 (7·2%) | 14 (9·7%) | 0·442 |
| **Risk factors and co-morbid conditions** |  |  |  |
| History of tobacco smoking, *(current/ former)* | 98 (64·5%) | 29 (20·1%) | < 0·001 |
| Use of biomass†, *yes* | 137 (90·1%) | 137 (95·1%) | 0·101 |
| Ever been treated for TB, *yes* | 38 (25·0%) | 24 (16·7%) | 0·078 |
| HIV status |  |  |  |
| Positive | 15 (9·8%) | 16 (11·1%) | 0·785 |
| Negative | 126 (82·9%) | 115 (79·9%) |  |
| Unknown | 11 (7·2%) | 13 (9·0%) |  |
| Nasal congestion or rhinorrhea, *yes* | 69 (45·4%) | 87 (60·4%) | 0·010 |
| Heart burn, *yes* | 86 (56·6%) | 82 (56·9%) | 0·949 |
| Body mass index (BMI), kg/m^2^, (median and IQR) |  |  |  |
| Underweight (BMI < 18·5 kg/m^2^), % | 87 (57·2%) | 78 (54·6%) | 0·001 |
| Normal weight (BMI 18·5 to 24·99 kg/m^2^), % | 40 (26·3%) | 18 (12·6%) |  |
| Overweight (BMI 25kg/m^2^ to 29·99 kg/m^2^), % | 17 (11·2%) | 25 (17·5%) |  |
| Obese (BMI ≥ 30 kg/m^2^), % | 8 (5·3%) | 22 (15·3%) |  |
| Hypertensive, i·e·, SBP/DBP cutoff 140/90 mmHg |  |  |  |
| SPO_2_ < 90, % | 18 (12·0%) | 27 (18·9%) | 0·102 |
| **Number of moderate/severe exacerbations (within the past one year)** |  |  |  |
| < 3 | 105 (69·5%) | 89 (61·8%) | 0·162 |
| ≥ 3 | 46 (30·5%) | 55 (38·2%) |  |
| Abbreviations used in the table: BMI: body mass index; COPD: chronic obstructive pulmonary diseases; DBP: diastolic blood pressure; FEV_1_: forced expiratory volume in the first second; FVC: forced vital capacity; IQR: interquartile range; LABA: long acting beta2 agonist; SBP: systolic blood pressure; TB: tuberculosis; SPO_2_: peripheral capillary oxygen saturation, %.  Biomass refers to wood and charcoal (typically used for cooking purposes). | | | |
