# Supplementary material for: Incidence and predictors of COPD mortality in Uganda: A 2-year prospective cohort study
Source: PLoS One. 2021 Feb 11;16(2):e0246850. doi: 10.1371/journal.pone.0246850 (PMC7877567; doi:10.1371/journal.pone.0246850)
Supplement: S1 Appendix — (PDF) [file pone.0246850.s002.pdf]

THE UGANDA REGISTRY FOR ASTHMA AND COPD (URAC) PROJECT

BASELINE DATA COLLECTION FORM

IDENTIFICATION

Patient's initials________________________

Hospital Number/clinic number _________

Date: __/__ /____

Study Identification Number_________

Address

Village/LCI________________________

District___________________________

Phone number________________________

Next of kin________________________

Relationship___________________________

Phone number, next of kin_____________________________

Patient's care facility at enrollment (circle)

1 Mulago Hospital chest clinic 2 Pulmonology ward 3 3 BEM/Casualty 4 Nsambya Hospital 5 Mengo

Hospital 6 Rubaga Hospital 7 Nakasero Hospital 8 International Hospital 9. Case Medical center

10 KADIC Hospital 11. Pediatric care clinic 12. Pediatric emergency 13. Pediatric ward 14. Other (specify) ________________________

Clinic diagnosis 1 Asthma 2 COPD

SOCIO-DEMOGRAPHIC AND RISK FACTORS

1. Age__________________________
2. Sex 1. Male 2. Female ☐
3. Marital status

   1Single 2 Married 3 Separated 4 Widowed ☐

4. District ________________________ Village_____________________
5. Specify tribe__________________________
6. Occupation ☐

   1. Unemployed 2. Housewife 3. Peasant farmer

   4. Business 5. Builder 6 Doctor 7 teacher/lecturer 8 lawyer 9 armed forces 10 engineer 11 student factory worker 12 nurse 13 allied health worker 14 Manger/Administrator 15

Clerical worker (e.g. sales clerk, secretaries, etc.) 8. Other (specify) ________________________

7. If employed, on average how much do you earn per month? ____________

8. Highest level of Education

1. None 2. Incomplete primary 3. Complete primary

4. Incomplete secondary 5. Complete secondary 6. Tertiary

RISK FACTORS

9. History of smoking

1. Yes 2. Former smoker 3 Never

If yes,

10. Estimated duration in months

11. Number of cigarettes/day

12. Are you in close contact with someone who smokes

1. Yes 2. No

13. If yes, estimated duration in months

Have you been exposed to biomass smoke

14. Wood?

1 Yes 2 No

15. Charcoal

1 Yes 2 No

16. If yes, years of exposure

17. Do use kerosene for lighting or cooking?

1 Yes 2 No

18. If yes, years of using kerosene

19. Have you ever been treated for TB?

1 Yes 2 No

20. HIV status

1 Positive 2 Negative 3 Unknown

21. Do any of your relatives suffer from asthma

1 Yes 2 No

22. Do you experience recurrent nasal congestion or rhinorrhea?

1 Yes 2 No

23. Do you experience heart burn/"acid irritation?

1 Yes 2 No

SYMPTOMS

24. Cough: do you cough several times most days?
1 Yes 2 No
If yes, duration in years

25. Sputum: do you bring up phlegm or mucus most days?
1 Yes 2 No

26. Wheezing: do you wheeze or have any whistling on the chest?
1 Yes 2 No

27. Shortness of breath: do you get out of breath more easily than others your age?
1 Yes 2 No

28. Chest Pain 1. Yes 2. No

29. Body swelling 1. Yes 2.No

30. Other 1. Yes 2.No
Specify ________________________

31. In the past one year, how many times did you have periods of breathing difficulty with increased cough with or without sputum **(<u>attacks</u>)?**

32. In the past one year how many times did you have to visit a health care facility because of respiratory problems

33. In the past one year how many times were you admitted because of respiratory problems

PHYSICAL EXAMINATION FINDINGS

34. Height
35. Weight
36. Respiratory rate
37. Pulse rate
38. Diastolic blood pressure
39. Systolic blood pressure
40. SPO2

SPIROMETRY
Pre-BD:

41. FVC (value/%)
42. FEV (value/%)

43. FEV_1/FVC ratio

Post-BD:

44. FVC (value/%)
45. FEV (value/%)
46. FEV_1/FVC ratio
47. Reversibility

Which of these medications have been used in the management of the patient?

1 Yes 2 No

48. Salbutamol tabs/syrup

49. Salbutamol inhaler

50. Aminophylline tablets

51. Aminophylline injections

52. Nebulized salbutamol

53. Nebulised salbutamol/ipratropium

54. Nebulized steroid

55. Oral steroids such prednisolone, dexamethasone

56. Injectable steroids such hydrocortisone, dexamethasone

57. Inhaled steroids such as beclomethasone inhaler

58. Combination inhalers (steroids and long acting beta agonists)

59. Combination inhaler (salbutamol/ipratropium)

60. Leukotriene modifiers such as monterlukast, zafirlukast

61. Antibiotics

62. Cough syrup or expectorant ☐

63. Ever used herbs for treatment of asthma/COPD ☐

64. If using herbs (specify)____

65. Others (specify)______________________________ ☐

66. Medication adherence score from Morisky 8-Item Medication Adherence Questionnaire attached

☐

COPD SECTION (COPD patients only)
MMRC breathlessness score

67. Which of the following statements best describes your situation? ☐

0 "I only get breathless with strenuous exercise"

1 "I get short of breath when hurrying on the level or walking up a slight hill"

2 "I walk slower than people of the same age on the level because of breathlessness or have to stop for breath when walking at my own pace on the level

3 "I stop for breath after walking about 100 yards or after a few minutes on the level

4 "I am too breathless to leave the house" or "I am breathless when dressing

CCQ (refer to questionnaire)

68. CCQ Total score ☐
69. Symptom score (number 1, 2, 5 and 6) ☐
70. Mental state score (number 3 and 4) ☐
71. Functional state score (number 7, 8, 9 and 10) ☐

72. 6MWT ☐

RISK REDUCTION

73. Have you been informed about stopping smoking ☐

1 Yes 2 No 3 NA

74. Have you been informed about reducing exposure to biomass smoke such as cooking in a separate kitchen

1 Yes 2 No 3 NA

75. If yes above how many years since you started cooking in a separate kitchen

ASTHMA SECTION

Which of the following make your asthma worse?

76. Upper respiratory infection such as sore throat, blocked nose, fever, cough, col
77. Exposure to household pets such as cats, dogs or poultry
78. Smoking or exposure to tobacco smoke when others are smoking
79. Strong emotions such as anger, excitement, anxiety
80. Cold weather
81. Drugs such those treating pressure and pain killers like aspirin
82. Exercise
83. Dust

84. Asthma control test score (from questionnaire)

BELIEFS ON ASTHMA

Answer 1 Yes 2 No

85. Asthma is a psychological condition

86. Asthma medicine is addictive

87. If I or my child takes asthma medicine every day for a long time, it will lose effectiveness and won't work when she's really sick

88. When I feel fine and have no symptoms it's because the asthma has gone away

89. Nebulizers are the best way to give asthma medicines

90. I can stop taking my medicine when I feel good and don't have any symptoms or problems breathing

91. If I or my child has asthma, I or he or she should not be allowed to play like other kids

92. Asthma inhalers weaken the heart and should not be used unless asthma is very serious

93. Herbs are effective in treating asthma

94. Asthma can be cured

95. Smoking does not trigger asthma or allergies

96. Inhaled steroids stunt children

97. What do you think causes asthma (describe)
