# Supplementary material for: Incidence and predictors of COPD mortality in Uganda: A 2-year prospective cohort study
Source: PLoS One. 2021 Feb 11;16(2):e0246850. doi: 10.1371/journal.pone.0246850 (PMC7877567; doi:10.1371/journal.pone.0246850)
Supplement: S2 Appendix — (PDF) [file pone.0246850.s003.pdf]

THE UGANDA REGISTRY FOR ASTHMA AND COPD (URAC) PROJECT

FOLLOW UP DATA COLLECTION FORM

IDENTIFICATION

Hospital Number/clinic number __________

Date: __/__ /____

Patient's initials_________________________

Study Identification Number__________

Patient's care facility at enrollment (circle)

1 Mulago Hospital chest clinic 2 Pulmonology ward 3 3 BEM/Casualty 4 Nsambya Hospital 5 Mengo Hospital 6 Rubaga Hospital 7 Nakasero Hospital 8 International Hospital 9. Case Medical center 10 KADIC Hospital 11. Pediatric care clinic 12. Pediatric emergency 13. Pediatric ward 14. Other (specify) _________________________

Clinic diagnosis 1 Asthma 2 COPD

Follow up interval 1 Month 6 2 Month 12 3 other

Follow up status 1 Attended 2 telephonic follow up 3 Dead 4 Lost to follow up (not seen for 2 consecutive months)

SYMPTOMS

1. **Cough**: do you cough several times most days? ☐
   1 Yes 2 No

   If yes, duration in years ☐☐☐
2. **Sputum**: do you bring up phlegm or mucus most days? ☐
   1 Yes 2 No
   ☐☐☐
3. **Wheezing**: do you wheeze or have any whistling on the chest? ☐
   1 Yes 2 No
4. **Shortness of breath**: do you get out of breath more easily than others your age?

   1 Yes 2 No ☐
5. **Chest Pain** 1. Yes 2. No ☐
   ☐☐☐

6. **Body swelling** 1. Yes 2.No
7. **Other** 1. Yes 2.No
   Specify ______________________

8. In the past one year, how many times did you have periods of breathing difficulty with increased cough with or without sputum **(<u>attacks</u>)?**

9. In the past one year how many times did you have to visit a health care facility because of respiratory problems

10. In the past one year how many times were you admitted because of respiratory problems

PHYSICAL EXAMINATION FINDINGS

11. Height

12. Weight

13. Respiratory rate

14. Pulse rate

15. Diastolic blood pressure

16. Systolic blood pressure
17. SPO2

SPIROMETRY
Pre-BD:
18. FVC (value/%)
19. FEV (value/%)
20. FEV_1/FVC ratio

Post-BD:

21. FVC (value/%)
22. FEV (value/%)
23. FEV_1/FVC ratio
24. Reversibility

Which of these medications have been used in the management of the patient?

1 Yes 2 No

25. Salbutamol tabs/syrup

26. Salbutamol inhaler ☐ ☐

27. Aminophylline tablets ☐

28. Aminophylline injections ☐

29. Nebulized salbutamol ☐

30. Nebulised salbutamol/ipratropium ☐

31. Nebulized steroid ☐

32. Oral steroids such prednisolone, dexamethasone ☐

33. Injectable steroids such hydrocortisone, dexamethasone ☐

34. Inhaled steroids such as beclomethasone inhaler ☐

35. Combination inhalers (steroids and long acting beta agonists) ☐

36. Combination inhaler (salbutamol/ipratropium) ☐

37. Leukotriene modifiers such as monterlukast, zafirlukast ☐

38. Antibiotics ☐

39. Cough syrup or expectorant ☐

40. Ever used herbs for treatment of asthma/COPD ☐

41. If using herbs (specify)____

42. Others (specify)_______________________________ ☐

COPD SECTION (COPD patients only)
MMRC breathlessness score

43. Which of the following statements best describes your situation? ☐

0 "I only get breathless with strenuous exercise"

1 "I get short of breath when hurrying on the level or walking up a slight hill"

2 "I walk slower than people of the same age on the level because of breathlessness or have to stop for breath when walking at my own pace on the level

3 "I stop for breath after walking about 100 yards or after a few minutes on the level

4 "I am too breathless to leave the house" or "I am breathless when dressing

CCQ (refer to questionnaire)

44. CCQ Total score ☐
45. Symptom score (number 1, 2, 5 and 6) ☐
46. Mental state score (number 3 and 4) ☐
47. Functional state score (number 7, 8, 9 and 10) ☐

48. 6MWT ☐

RISK REDUCTION

49. Have you been informed about stopping smoking ☐

1 Yes 2 No 3 NA

50. Have you been informed about reducing exposure to biomass smoke such as cooking in a separate kitchen ☐

1 Yes 2 No 3 NA

51. If yes above how many years since you started cooking in a separate kitchen ☐☐

ASTHMA SECTION

Which of the following make your asthma worse?

52. Upper respiratory infection such as sore throat, blocked nose, fever, cough, co[illegible] ☐
53. Exposure to household pets such as cats, dogs or poultry ☐
54. Smoking or exposure to tobacco smoke when others are smoking ☐
55. Strong emotions such as anger, excitement, anxiety ☐
56. Cold weather ☐

57. Drugs such those treating pressure and pain killers like aspirin

58. Exercise

59. Dust

60. Asthma control test score (from questionnaire)
